# Impact of South Korea’s Comprehensive Nursing Service Policy on Nurse and Patient Outcomes

**DOI:** 10.3390/healthcare8030223

**Published:** 2020-07-22

**Authors:** Seon Heui Lee, Soyoung Yu, Miok Kim, Hee Sun Kim

**Affiliations:** 1 Department of Nursing Science, College of Nursing, Gachon University, Incheon 21936, Korea; sunarea87@gachon.ac.kr; 2 College of Nursing, CHA University, Gyeongghi-do Pocheon 120, Korea; 3 Department of Nursing, College of Medicine, Dankook University, Cheonan 31116, Korea; aprilsea@hanmail.net; 4 College of Nursing, Jeonbuk National University, Jeonju 54896, Korea; joha0219@naver.com

**Keywords:** comprehensive nursing service, health policy, nursing outcomes, patient outcome

## Abstract

In some Asian and African countries, caregivers of patients are permitted to reside in hospital rooms and support the daily tasks of patient care. To solve the various problems that this system could cause, the Korean government has established a comprehensive nursing service, whereby caregivers are no longer permitted in the hospital and, instead, nurses provide all the patient care. This study aimed to identify and evaluate the effectiveness of the comprehensive nursing service, by surveying 1348 nurses to evaluate nursing outcomes—specifically, the Nurses’ Assessment of Quality Scale, job satisfaction, and turnover intention. A total of 396 patients were also surveyed to determine patient outcome, in particular patient satisfaction. In the comprehensive nursing service ward, the total score on the Nurses’ Assessment of Quality Scale, job satisfaction, and patient satisfaction scores were higher than in the non-comprehensive nursing service ward. Moreover, turnover intention was lower. All differences were statistically significant. The results of this study demonstrate that the decision to implement policy-based comprehensive nursing services has thus far been beneficial. In the future, the government should revise and supplement its policies through various socioeconomic assessments.

## 1. Introduction

Nursing care delivery systems vary depending on the national policies, socioeconomic environment, and health care systems in each country. The difference in hospital culture between South Korea and countries such as the United States, United Kingdom, Germany, and France is that the latter strictly restrict hospital visiting times by family members and caregivers, which are firmly established by the nurse-centered nursing system. In general, both formal and informal patient caregivers in low-income and certain other countries are intimately involved in patient care and often live on the hospital grounds [1]. In addition to South Korea, many Asian countries, including China and Taiwan, and some African countries allow caregivers such as family members to reside in hospital rooms to support the daily tasks of patient care [2]. South Korea has maintained this patient–individual caregiver nursing system for the past 70 years. It is a system in which caregivers (typically family members) live in the hospital during the hospitalization period, enabling a small number of nurses to care for a larger number of hospitalized patients, as compared to Western countries. However, the form of patient care in hospitals has changed from care by family members to care by paid caregivers [3]. Approximately 40% of inpatients in Korea are now cared for by paid caregivers who are employed directly by the patients [4]. The cost of hiring such caregivers has increased indirect medical expenses and placed an economic burden on patients [5]. Thus, with the aim of solving these problems, since May 2010, the Korean government has invested KRW 2.4 billion from the government budget and KRW 2 billion from the budget of the health insurance corporation to conduct a pilot project in 10 hospitals. In July 2013, 13 medical institutions implemented the first pilot project, which required hospitals to disallow individual caregivers [6]. In February 2014, the project began its second phase and includes 20 more public hospitals; the name of the project was also changed to the “comprehensive nursing service.” In the comprehensive nursing service project, all inpatient nursing services are provided by assigning appropriate nursing personnel to patients, without any individual caregivers. The number of beds covered by the comprehensive nursing and caregiving service doubled from 7000 in 2015 to 15,000 in 2016; then, as it extended its nationwide reach, the service reached 23,000 beds in 2017. In 2019, the government further expanded the number of comprehensive nursing service hospitals, which now include not only national and public hospitals but also private ones. However, it is true that in the early days of the government-led initiative, before the nationwide expansion, there were several concerns about this policy. For example, a typical concern was whether a nurse could care sufficiently for a patient in the absence of a caregiver, either formal or informal, with regard to patient safety and treatment outcomes [7]. Assessment of the new nursing care delivery system can be classified into two categories: the impact of the policy on the user’s side and that on the supplier’s side. Patient satisfaction with the new system is a concern of the users, whereas nurse performance, job satisfaction, and turnover intention are concerns of the suppliers [8]. It is noteworthy that this new nursing care delivery system would change the scope of practice of the nursing profession. For example, nurses directly assist and care for patients in all activities of daily living. Nurses who provide comprehensive nursing services feel that the scope of their work and role is not well delineated or clear; hence, they experience “role conflict” due to their simultaneous rendering of caregiver services, and feel burdened by excessive work [9]. Low job satisfaction makes it difficult for nurses to perform their tasks efficiently and positively, and leads to poor quality of patient care and an increased nurses’ turnover rate [10]. The problem of job satisfaction among nurses who provide comprehensive nursing services can obstruct the steady expansion and institutionalization of comprehensive nursing service hospitals by making the supply of nurses scarce and their management difficult.

**Figure 1:**
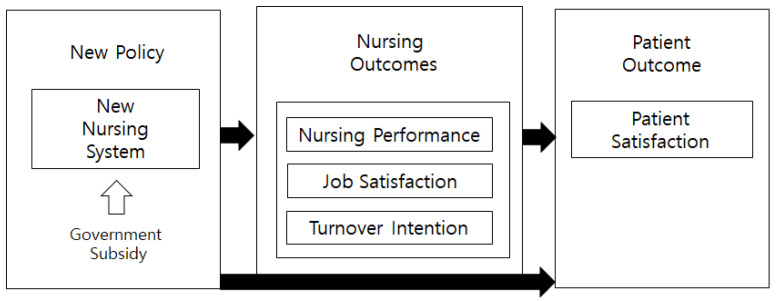
Conceptual framework of this study.

Based on the above, this study aimed to identify and evaluate the effectiveness of the comprehensive nursing service. The study hypothesized that there is no difference in the indicators of nurses and patients between the comprehensive nursing service ward and the non-comprehensive nursing service ward. Specifically, it attempted to evaluate nursing outcomes such as nursing performance, job satisfaction, and turnover intention, and the patient outcome, patient satisfaction (Figure 1). Evaluation of the comprehensive nursing service would enable countries implementing this policy to verify the efficacy of nursing services through trial and error in other countries like South Korea. Evaluation would also help to confirm the strengths and weaknesses of the system for countries that have not yet implemented it.

## 2. Methods

### 2.1. Study Setting

A survey was administered to 1500 nurses who had consented to participate. The survey identified the indicators of nursing outcomes to assess the effectiveness of the comprehensive nursing service. The participants comprised 700 nurses in the general ward and 800 nurses in the comprehensive nursing service ward from 45 hospitals (17 public and 28 private) in South Korea. The difference between the two nursing systems was evaluated.

A survey of patient satisfaction was conducted on 450 patients (comprehensive: 250; non-comprehensive ward: 200) hospitalized at the same 10 hospitals and wards out of 45 hospitals that surveyed nurses (seven private and three public hospitals) who were implementing comprehensive care services. To minimize the influence of differences of characteristics (for example, clinical department, medical diagnosis, number of hospital days) between comprehensive and non-comprehensive nursing service wards, the medical institution requested the hospital to select a non-comprehensive ward with characteristics similar to those of the comprehensive nursing service ward.

### 2.2. Data Collection

Data were collected from nurses of 45 hospitals, including general and tertiary hospitals, which granted approval for the study through their nursing departments. The inclusion criteria for hospitals in this study were that the comprehensive nursing service ward in the hospital must have been functional for more than three months and the hospital must agree to participate in the research. The exclusion criteria were hospitals that were not operating the comprehensive nursing service ward or that had been operating it for less than three months. The questionnaire form informed participants on the confidentiality of their responses and the voluntary nature of participation in the study. It also stated the study purpose and method, and requested their consent to participate. Respondents were allowed to discontinue the survey at any time if they so wished, without any consequences. After informing the participants of the study purpose, those who understood the purpose and agreed to participate completed the questionnaire. The members of the research team who were trained to compile the questionnaire collected data between July and August 2015. The Institutional Review Board approved the overall study protocol (IRB NO. 1044396-201504-HR-026-01).

### 2.3. Measures

The Nurses’ Assessment of Quality Scale (Acute Care Version, NAQS-ACV): Nursing performance was measured using the Assessment of Nursing Care Scale developed by Lynn et al. [11]. This instrument was derived from qualitative interviews with acute care staff nurses under the question: “how would you describe or define quality nursing care?” Therefore, it is a tool which allows nurses to self-assess the quality of care they provide, rather than evaluating the quality of care from the patient’s point of view. The first page of the questionnaire asks respondents to “think about a patient that you recently cared for and remember well.” The scale was translated and used by the researchers after obtaining permission from the developer. The instrument consisted of 77 questions divided into three categories and eight areas. The three categories consisted of personal factors related to the quality of nursing services to determine the volume of nursing services being performed, the work environment, and whether the nurses’ own personal factors affect patient care. The eight areas were: vigilance, advocacy, individualization, interaction, work environment, unit collaboration, personal characteristics, and mood. The questions were answered using a 4-point Likert scale, ranging from 1 (strongly disagree) to 4 (strongly agree). A higher score indicated better quality of nursing service performance. Reliability estimates for the factors ranged from 0.74 to 0.94 at the time of development [11]. In this study, the Cronbach’s alpha was 0.94.

Job Satisfaction: The Job Satisfaction Index developed by Stamps et al. [12] and modified by Bae [13] was used in this study. This tool consisted of seven sub-categories, comprising pay level, professional positions, administrative affairs, autonomy, job requirements, interaction, and relations with nurses and doctors or related departments. Each of the total 20 questions was answered on a 5-point scale, ranging from 1 (strongly disagree) to 5 (strongly agree). The negative questions were reverse scored. A higher score indicated a higher level of job satisfaction. At the time of development of this tool, reliability was 0.76. In this study, the Cronbach’s alpha was 0.78.

Turnover Intention: Kim [14] modified and supplemented the instrument for measuring turnover intention developed by Mobley [15]. It consisted of six questions, answered using a 5-point Likert scale, ranging from 1 (strongly agree) to 5 (strongly disagree). A higher score indicated a higher degree of turnover. At the time of development of this tool, reliability was 0.76. In this study, the Cronbach’s alpha was 0.75.

Patient Satisfaction: The Patient Satisfaction Index developed by Lee [16] was used. It consisted of 35 questions, assessed on a 5-point scale, and covered five areas: physical nursing satisfaction, therapeutic nursing satisfaction, environmental nursing satisfaction, emotional nursing satisfaction, and information nursing satisfaction. A higher score indicated a higher level of patient satisfaction for nursing performance. At the time of development of this tool, reliability was 0.93. In this study, the Cronbach’s alpha was 0.95.

### 2.4. Data Analysis

The data were analyzed using SPSS WIN 25.0 (SPSS Inc., Chicago, IL, USA). The general characteristics of the nurses and the patients of the comprehensive nursing service ward were analyzed using numbers, percentages, means, and standard deviations. The analysis was performed using Chi-square test. The average difference was analyzed by an independent t-test and analysis of variance (ANOVA).

## 3. Results

Of the 1500 registered nurses who were invited to participate in the survey, 1348 agreed and completed the questionnaire (response rate of 89.8%). Their data were used in the analysis. Of the 450 patients approached for the survey, 396 patients agreed and completed the questionnaire (response rate of 88%).

### 3.1. Indicators of Nursing Outcomes

The mean age of nurses in comprehensive and non-comprehensive nursing service wards was 29.38 ± 10.61 years and 29.52 ± 6.96 years, respectively. The average length of nurses’ clinical experience was 71.49 ± 78.65 months and 78.21 ± 78.53 months in comprehensive and non-comprehensive nursing service wards, respectively. Among the nurses in comprehensive nursing service wards, 501 had completed a three-year college degree, 266 had completed a four-year university degree, and 26 had completed a master’s degree or higher. Among the nurses in non-comprehensive nursing service wards, 337 had completed a three-year college degree, 198 had completed a four-year university degree, and 18 had completed a master’s degree or higher. The homogeneity of the two groups was thus identified (Table 1).

The nursing performance score, job satisfaction among nurses, and turnover intention were analyzed as nursing outcome indicators. As shown in Table 2, differences in nursing performance were measured using the Assessment of Nursing Care Scale. The NAQS-ACV score was 2.98 for comprehensive nursing service wards and 2.91 for non-comprehensive nursing service wards. There was a statistically significant difference between the scores (*p* < 0.0001). Among the sub-items, the two groups differed significantly in the “vigilance” and “work environment” factors.

The difference in nursing outcome indicators (i.e., job satisfaction and turnover intention of nurses) between the two wards is presented in Table 3. Job satisfaction was higher (*p* < 0.001) and turnover intention (*p* = 0.02) was lower in the comprehensive than in the non-comprehensive nursing service wards.

### 3.2. Indicators of Patient Outcome

The mean age of patients was 59.26 ± 17.67 years and 45.99 ± 22.65 years in comprehensive and non-comprehensive nursing service wards, respectively. The homogeneity of the two groups was identified based on patients’ admission period, and data on whether a patient had undergone surgery during hospitalization in the ward where s/he was hospitalized (Table 4).

The differences in patient outcome are shown in Table 5. In this study, patient outcome was assessed by measuring patient satisfaction levels. Patient satisfaction score was higher in the comprehensive than in the non-comprehensive nursing service wards (*p* < 0.0001). Patient satisfaction scores for “Physical (*p* < 0.0001),” “Therapeutic (*p* < 0.0001),” “Environmental (*p* < 0.0001),” “Emotional (*p* < 0.0001),” and “Informational (*p* = 0.0001)” nursing care were significantly higher in the comprehensive than in the non-comprehensive nursing service wards. Based on these findings, the study hypothesis was rejected.

The general characteristics of comprehensive nursing care service ward affecting patient satisfaction are as shown in Table 6. There was no difference in patient satisfaction according to gender, age, occupation, hospitalization period, and the ratio of nurses to patients in the comprehensive nursing care service ward.

## 4. Discussion

This study aimed to evaluate the effectiveness of the comprehensive nursing service as a new nursing care model, specifically in terms of nursing performance, job satisfaction, and turnover intention as nursing outcomes, and patient satisfaction as the patient outcome. This study has significance in policy evaluation in that it confirms the effectiveness of the policy formulated by the Korean government, which has increased the number of hospitals that have been implementing the comprehensive nursing service since 2010. This study is also important because it targeted a large number of hospitals, nurses, and patients to collect data to evaluate the new vis-à-vis the old nursing care system. The latter has been implemented in Korea for more than 70 years.

The results of the NAQS-ACV showed differences between comprehensive and non-comprehensive nursing service wards in terms of nursing outcomes. The NAQS-ACV was created based on qualitative data collected through the interview questions regarding what qualitative nursing is [11]. This self-assessment nursing tool evaluates nurse performance. The evaluation of the new nursing system by nurses themselves, who play the role of caregiver, is meaningful.

Although direct comparisons with previous studies are difficult since there has been no research on comprehensive nursing service wards using the NAQS-ACV, a previous study did analyze the importance and quality of the work performed in comprehensive nursing service wards [17]. According to Lee [17], nurses who provide comprehensive nursing services have a high level of indirect nursing performance. They recognize that physiological nursing is important and prioritize it. Their performance is reportedly poorer because they perform relatively lower volumes of psychosocial nursing work. On the contrary, this study showed that nurses from the comprehensive nursing service ward, including those performing psychosocial nursing care, are likely to be more interested in a patient’s condition than those in non-comprehensive nursing service wards (see the individualization factor in Table 2). Lee [17] indicated that the priority given to psychological nursing care is relatively lower than that given to physiological care. In this study, the comparison of the two wards showed that the comprehensive nursing service wards are more focused on individual patients. Meanwhile, the sub-items that did not show any significant difference between the two wards were related to the cooperation system of the ward to which the nurse belongs (unit collaboration) and whether the nurse’s mood and fatigue affect patient care (mood). This is because there is no large difference in the cooperation system between the comprehensive nursing service and the general wards. Therefore, the result may be attributed to the nurses’ belief that there is no difference between the two wards, regardless of the kind of nursing service provided.

The study found differences in nursing performance, job satisfaction, and turnover intention between comprehensive and non-comprehensive nursing service wards. Previous studies have also found differences in nurses’ job satisfaction. Some studies reported that nurse satisfaction was lower in the comprehensive nursing service wards [17,18,19]; others showed a lack of difference among nurses between the two wards [20,21]; while yet another study reported higher nurse satisfaction in comprehensive service wards [22].

The results of a previous study demonstrated that patient satisfaction with the nursing service and hospital reuse intention, among other factors, was positive [23]. Many studies have evaluated patient satisfaction with nursing service in the ward implementing comprehensive nursing service and compared it with patient satisfaction with nursing in the general ward [7,24,25]. The results have demonstrated that patients are satisfied with the comprehensive nursing service. However, indicators related to the nurses’ viewpoint, specifically in job satisfaction and turnover intention, have been reported differently in different studies. Therefore, it is necessary to pay attention to nurses’ job satisfaction. The comprehensive nursing service is a good model to regulate the burden related to patient care and provide high quality nursing, as confirmed through the results of this study. However, the key to establishing this service properly and expanding its scope is to hire competent nursing staff. As decreased job satisfaction and high stress can lead to high turnover rates among nurses, it is necessary to address the problems identified by this evaluation [23], including excessive workloads and ambiguous role-sharing among nursing staff. Therefore, it is crucial to create satisfactory working conditions for nurses [26]. When nurses are satisfied with their work, the quality of nursing services provided to patients as well as patient safety can be guaranteed. In South Korea in particular, there are many difficulties with providing quality nursing care due to the high ratio of patients to nurses. The comprehensive nursing service prevents the ratio of patients to nurses from exceeding a certain standard, making it a nursing delivery model with many long-term advantages in terms of creating a healthier work environment for nurses [20,26]. Therefore, nursing managers should take active interest in the effects of the expansion of the comprehensive nursing service, such as holding research or reporting sessions at the nurses’ association level, so that nurses’ voices can be reflected in national policies.

In an international context, studies were conducted to assess the role of hospital caregivers [2] and their needs in the application of person-centered care [1]. Particularly in countries with a low medical-personnel-to-population ratio, the necessity for inpatient caregivers is highlighted [2]. For long-term medical progress at the national level, including professional patient management, it is necessary to institute a policy that does not impose responsibility for inpatient care on caregivers. The viability of such a policy will depend on the socioeconomic environment of each country, including indices such as the proportion of single-person households and population aging, but the government must gradually push the policy so that hospitals can take full responsibility for inpatient care. Inpatients, on the other hand, should steadily advocate such a policy to ensure that they can avail an adequate level of care. This new nursing care policy must be evaluated longitudinally in a variety of aspects, namely the pros and cons from a socioeconomic perspective as well as from the perspective of patients and nurses as analyzed in this study. For instance, it is necessary to examine the change in socioeconomic costs following implementation of the comprehensive nursing service. Moreover, the policy subsidy for the comprehensive nursing service, which is currently supported by the Korean government, should be continued until the policy is stabilized.

## 5. Limitations

The limitations of this study are as follows: (1) a self-report questionnaire was used to examine the quality of care provided; (2) among the various nurse-related indicators, only job satisfaction and turnover intention were evaluated, while only patient satisfaction was assessed as patient outcome. Future studies must examine other aspects.

## 6. Conclusions

This evaluation of the effectiveness of the comprehensive nursing service as a new nursing care model, focusing on outcomes for both nurses and patients, provides added value to health care policy makers and the public. The results of this study demonstrated that conditions were better in the comprehensive nursing service ward than in the general ward. In the comprehensive nursing service ward, the total score on the Nurses’ Assessment of Quality Scale was higher than that in the non-comprehensive nursing service ward. Job satisfaction was higher in the comprehensive nursing service ward and turnover intention was lower. The patient satisfaction scores in the comprehensive nursing service ward were higher than in the non-comprehensive nursing service ward. This finding supports the national policy on the comprehensive nursing service, which the Korean government has expanded since 2015 despite many socioeconomic issues. To confirm the supplementary points of the current policy and make the comprehensive nursing service more effective for the changing society, further evaluations of the socioeconomic aspects of the policy should be conducted. For example, it is necessary to compare and evaluate the nursing methods applied in other countries and reflect them in local policy settings.

## Figures and Tables

**Table 1:** Characteristics of Participating Nurses (*n* = 1348).

Characteristic	Comprehensive Nursing Service Ward (*n* = 795)	Non-Comprehensive Nursing Service Ward (*n* = 553)	Chi-Square or t (*p*)
	*n* (%) or Mean ± SD	*n* (%) or Mean ± SD	
Age (years)	29.38 ± 10.61	29.52 ± 6.96	−0.26 (0.790)
Clinical Experience (Month)	71.49 ± 78.65	78.21 ± 78.53	−1.54 (0.123)
Education			
Three-year college degree	501 (63.2)	337 (60.9)	0.75 (0.689)
Four-year university degree	266 (33.5)	198 (35.8)	
Graduate school or higher	26 (3.3)	18 (3.3)	

SD: Standard Deviation.

**Table 2:** Scores on Nurses’ Assessment of Quality Scale (Acute Care Version, NAQS-ACV) (*n* = 1348).

Category	Factor	Comprehensive Nursing Service Ward (*n* = 795)	Non-Comprehensive Nursing Service Ward (*n* = 553)	t	*p*
		Mean (SD)	Mean (SD)		
Total (min 1–max 4)		2.98 (0.25)	2.91 (0.2)	5.65	<0.0001
Section 1	Vigilance	2.96 (0.34)	2.85 (0.32)	5.85	<0.0001
	Advocate	3.13 (0.29)	3.08 (0.24)	3.13	0.0018
	Individualization	2.95 (0.34)	2.89 (0.3)	3.34	0.0008
	Interaction	3.01 (0.3)	2.96 (0.24)	3.29	0.001
Section 2	Work environment	2.75 (0.37)	2.59 (0.38)	7.65	<0.0001
	Unit collaboration	2.93 (0.33)	2.92 (0.33)	0.88	0.3779
Section 3	Personal characteristics	3.17 (0.38)	3.1 (0.34)	3.61	0.0003
	Mood	2.96 (0.49)	2.96 (0.47)	0.15	0.882

SD: Standard Deviation.

**Table 3:** Job Satisfaction and Turnover Intention among Nurses (*n* = 1348).

Indicator	Comprehensive Nursing Service Ward (*n* = 795)	Non-Comprehensive Nursing Service Ward (*n* = 553)	t	*p*
	Mean (SD)	Mean (SD)		
Job Satisfaction (min 1–max 5)	3.22 (0.38)	3.16 (0.38)	2.91	<0.001
Turnover intention (min 1–max 5)	3.27 (0.52)	3.34 (0.54)	−2.40	0.02

SD: Standard Deviation.

**Table 4:** Descriptive Statistics of General Characteristics of Patients (*n* = 396).

Characteristics	Comprehensive Nursing Service Ward (*n* = 238)	Non-Comprehensive Nursing Service Ward (*n* = 158)	t/F (*p*)
	*n* (%) or Mean ± SD	*n* (%) or Mean ± SD	
Age (years)	59.26 ± 17.67	45.99 ± 22.65	6.36 (0.00)
Admission Period (days)			5.62 (0.13)
0–6 ^a^	74 (38.5)	40 (33.1)	
7–13 ^b^	52 (27.1)	24 (19.8)	
14–27 ^c^	36 (18.8)	34 (28.1)	
≥28 ^d^	30 (15.6)	23 (19.0)	
Surgical procedure			
NO	160 (67.2)	114 (72.1)	
YES	78 (32.8)	44 (27.9)	1.08 (0.29)

SD: Standard Deviation.

**Table 5:** Patient Satisfaction Scores (*n* = 396).

Category	Comprehensive Nursing Service Ward (*n* = 238)	Non-Comprehensive Nursing Service Ward (*n* = 158)	*t*	*p*
	Mean (SD)	Mean (SD)		
Total score (min 1–max 5)	4.34 (0.63)	4.01 (0.63)	5.14	<0.0001
Physical Nursing Satisfaction	4.34 (0.7)	3.92 (0.67)	5.97	<0.0001
Therapeutic Nursing Satisfaction	4.44 (0.61)	4.18 (0.66)	4	<0.0001
Environmental Nursing Satisfaction	4.38 (0.75)	3.89 (0.9)	5.77	<0.0001
Emotional Nursing Satisfaction	4.31 (0.65)	3.97 (0.67)	5.06	<0.0001
Informational Nursing Satisfaction	4.26 (0.74)	3.97 (0.71)	3.88	0.0001

SD: Standard Deviation.

**Table 6:** Differences of Patient Satisfaction Scores according to the General Characteristics of Comprehensive Nursing Care Service Ward (*n* = 238).

Characteristics	Category	*n* (%)	Patient Satisfaction Scores	
			Mean (SD)	t/F (*p*)
Gender	Male	112 (49.1)	4.36 (0.61)	0.475 (0.63)
	Female	116 (50.9)	4.32 (0.67)	
Age (years)	≤39 ^a^	31 (13.0)	4.42 (0.66)	0.502 (0.68)
	40~49 ^b^	46 (19.3)	4.29 (0.7)	
	50~59 ^c^	43 (18.1)	4.35 (0.6)	
	60~69 ^d^	40 (16.8)	4.39 (0.54)	
	≥70 ^e^	78 (32.8)	4.31 (0.66)	
Occupation	No	148 (64.9)	4.31 (0.65)	0.709 (0.40)
	Yes	80 (35.1)	4.39 (0.62)	
Admission period	0–6 ^a^	74 (38.5)	4.32 (0.69)	0.101 (0.95)
	7–13 ^b^	52 (27.1)	4.32 (0.57)	
	14–27 ^c^	36 (18.8)	4.26 (0.73)	
	≥28 ^d^	30 (15.6)	4.32 (0.62)	
Nurse to Patient ratio	1:7 ^a^	20 (8.4)	4.41 (0.39)	0.296 (0.88)
	1:7.5 ^b^	59 (24.8)	4.27 (0.62)	
	1:8 ^c^	20 (8.4)	4.36 (0.48)	
	1:10 ^d^	117 (49.1)	4.34 (0.70)	
	1:12 ^e^	22 (9.24)	4.41 (0.54)	
